# EBI Metagenomics in 2017: enriching the analysis of microbial communities, from sequence reads to assemblies

**DOI:** 10.1093/nar/gkx967

**Published:** 2017-10-23

**Authors:** Alex L Mitchell, Maxim Scheremetjew, Hubert Denise, Simon Potter, Aleksandra Tarkowska, Matloob Qureshi, Gustavo A Salazar, Sebastien Pesseat, Miguel A Boland, Fiona M I Hunter, Petra ten Hoopen, Blaise Alako, Clara Amid, Darren J Wilkinson, Thomas P Curtis, Guy Cochrane, Robert D Finn

**Affiliations:** EMBL-EBI European Bioinformatics Institute, Wellcome Trust Genome Campus, Hinxton, Cambridge CB10 1SD, UK; School of Mathematics & Statistics, Newcastle University, Newcastle upon Tyne NE1 7RU, UK; School of Civil Engineering and Geosciences, Newcastle University, Newcastle upon Tyne NE1 7RU, UK

## Abstract

EBI metagenomics (http://www.ebi.ac.uk/metagenomics) provides a free to use platform for the analysis and archiving of sequence data derived from the microbial populations found in a particular environment. Over the past two years, EBI metagenomics has increased the number of datasets analysed 10-fold. In addition to increased throughput, the underlying analysis pipeline has been overhauled to include both new or updated tools and reference databases. Of particular note is a new workflow for taxonomic assignments that has been extended to include assignments based on both the large and small subunit RNA marker genes and to encompass all cellular micro-organisms. We also describe the addition of metagenomic assembly as a new analysis service. Our pilot studies have produced over 2400 assemblies from datasets in the public domain. From these assemblies, we have produced a searchable, non-redundant protein database of over 50 million sequences. To provide improved access to the data stored within the resource, we have developed a programmatic interface that provides access to the analysis results and associated sample metadata. Finally, we have integrated the results of a series of statistical analyses that provide estimations of diversity and sample comparisons.

## INTRODUCTION

The term ‘metagenomics’ describes the collective analysis of microbial genomes sampled from a particular environment, such as soil, seawater or a human body site. Such genomic analyses can provide powerful insights into microbial community composition and function. Underpinned by dramatically falling DNA sequencing costs, metagenomic analyses have become increasingly mainstream in recent years and have been applied to a diverse variety of fields, including marine ecology, agriculture, food manufacture, bioenergy production and human health. Notable recent metagenomic studies have helped to identify new microbial *phyla* with distinct metabolic repertoires (1,2), to discover novel CRISPR–Cas systems (3) with potential application in gene editing, to rapidly expand the library of known protein structures (4), and to link the microbiome to a host of disorders, from rheumatoid arthritis (5) to Parkinson's Disease (6).

Despite widespread interest and uptake by the research community, metagenomic analyses can be challenging due to the volume and nature of the underlying sequence data. Single metagenomic whole genome shotgun (WGS) sequencing runs can yield hundreds of millions of sequences, representing tens of gigabytes (GB) of uncompressed data on disk. With many experiments involving tens or hundreds of such runs, data volumes can quickly overwhelm the storage capacities and analysis capabilities of individual researchers. The sequences themselves, meanwhile, tend to be relatively short, ranging from approximately 100 to 500 bp (with a mean of ∼230 base pairs) following merging and quality trimming for typical Illumina paired-end runs - the dominant sequencing platform for metagenomics. This can pose a problem when trying to determine the functional activity encoded within a metagenome. Typically, short reads represent only small fragments of the underlying full-length gene sequences. Predicted protein coding sequences based on these fragments often lack the distinguishing features of full length proteins, such as binding sites, active sites, or other important amino acid motifs that can be used to infer function.

Assembly of sequences *in silico* into longer contigs helps to address this problem, allowing more detailed functional annotation. In addition, the generation of longer assemblies enables detection of larger and more complex genomic features, such as operons and CRISPRs, and allows inference of function based upon genome context. It also potentially allows taxonomic binning of contigs, and partial, or even complete, reconstruction of genomes (7,8). However, many of the best-performing assembly tools require substantial computational resources. For example, assembly of a soil WGS run with 16 GB paired-end gzipped fastq files from Alaskan Tundra (9) (ENA study accession PRJEB10725, run accession ERR1035438) requires over 500GB RAM to assemble using metaSPAdes (10) version 3.10.1. Not all researchers have access to this kind of computational power.

Another common problem is the effect that different sequence processing tools, analysis software and reference databases exert upon analysis results. Differences in algorithms and/or parameters at each sequence processing stage, from initial quality control (QC) (11), to gene prediction (12,13), through to assembly (14), can all substantially impact the number, quality and average length of sequences in a metagenomic dataset. Alongside this, choice of analysis tools, reference databases, and software settings can profoundly influence taxonomic classification and function prediction (15–17). As a result, it is hard to make meaningful comparisons between the analysis results of two different datasets that have been processed using different pipelines.

EBI Metagenomics (https://www.ebi.ac.uk/metagenomics/) aims to address many of these issues as a freely available hub for the analysis, exploration and archiving of metagenomic data. In common with analysis platforms such as MG-RAST (18) and IMG/M (19), EBI Metagenomics provides standardised processing and analysis pipelines that allow functional and taxonomic analyses of user-submitted sequences. It also offers a variety of analytical and visualization tools to support examination and comparison of datasets. Through partnership with the European Nucleotide Archive (ENA), EBI Metagenomics also has a unique archiving remit. Datasets submitted for analysis are accessioned and stored permanently within ENA (which operates under the International Nucleotide Sequence Database Collaboration (INSDC)) for public reference.

EBI Metagenomics supplements its analysis of user-submitted data by processing publicly available metagenomic datasets drawn from the ENA. Enabling large scale data analysis using standardised pipelines allows new user-submitted studies to be placed in context with other data (e.g. does a marine study drawn from a particular location have the same microbial community structure as a similar study performed by another research group), increasing data reuse and maximizing the knowledge that can be extracted from both datasets. The strategy of pursuing publicly available data has led EBI Metagenomics to grow rapidly over the last 2 years to become one of the world's largest metagenomic data repositories. It currently houses over 100 000 publicly available datasets, sampled from a wide range of environments, ranging from insect digestive tracts to hydrothermal vents.

Here, we report a number of significant new developments with EBI Metagenomics over the last two years, including two new analysis pipeline updates, an upgrade to the taxonomic analysis component to enable eukaryotic classification, along with a 10-fold increase in the number of datasets analysed by the resource. In addition, we report the development of a detailed web-based faceted search facility, underpinned by extensive indexing of contextual metadata, and a first iteration of a new RESTful API to allow programmatic access to metadata and analysis results. We also discuss the resource's recent move towards provision of assembly of metagenomic datasets and the concomitant development of a searchable non-redundant metagenomic peptide database, representing 10s of millions of novel sequences.

## UPDATES TO DATA CONTENT

‘Metagenomics’ is often used as a catch-all term and can refer to several different experiment types. Within EBI Metagenomics, data is divided into WGS sequencing of DNA extracted from environmental samples (**metagenomics**), whole transcriptome shotgun sequencing (**metatranscriptomics**) and metagenetic studies that target markers, such as the small subunit (SSU) ribosomal ribonucleic acid gene (**amplicon**) or other genes (**metabarcoding**), such as ITS1 or COX1, in order to assign taxonomy. In addition, the resource provides analysis of user-submitted assembled sequence data (**assembly**). In a recent change to our pipeline capability (outlined in the **Assembly and peptide database creation** section below), EBI Metagenomics has begun to offer assembly of public metagenomes and user-submitted data upon request.

Analysed data in EBI Metagenomics are structured into projects, samples and runs, mimicking the organisation found in ENA. These are arranged in potential one-to-many relationships, where one project may contain several samples that can each have a number of associated runs. Such multiple runs can take the form of technical replicates or different experiments performed upon the same sample material (eg, metagenomic analysis of WGS data, plus analysis of the assembled underlying reads).

**Figure 1.**
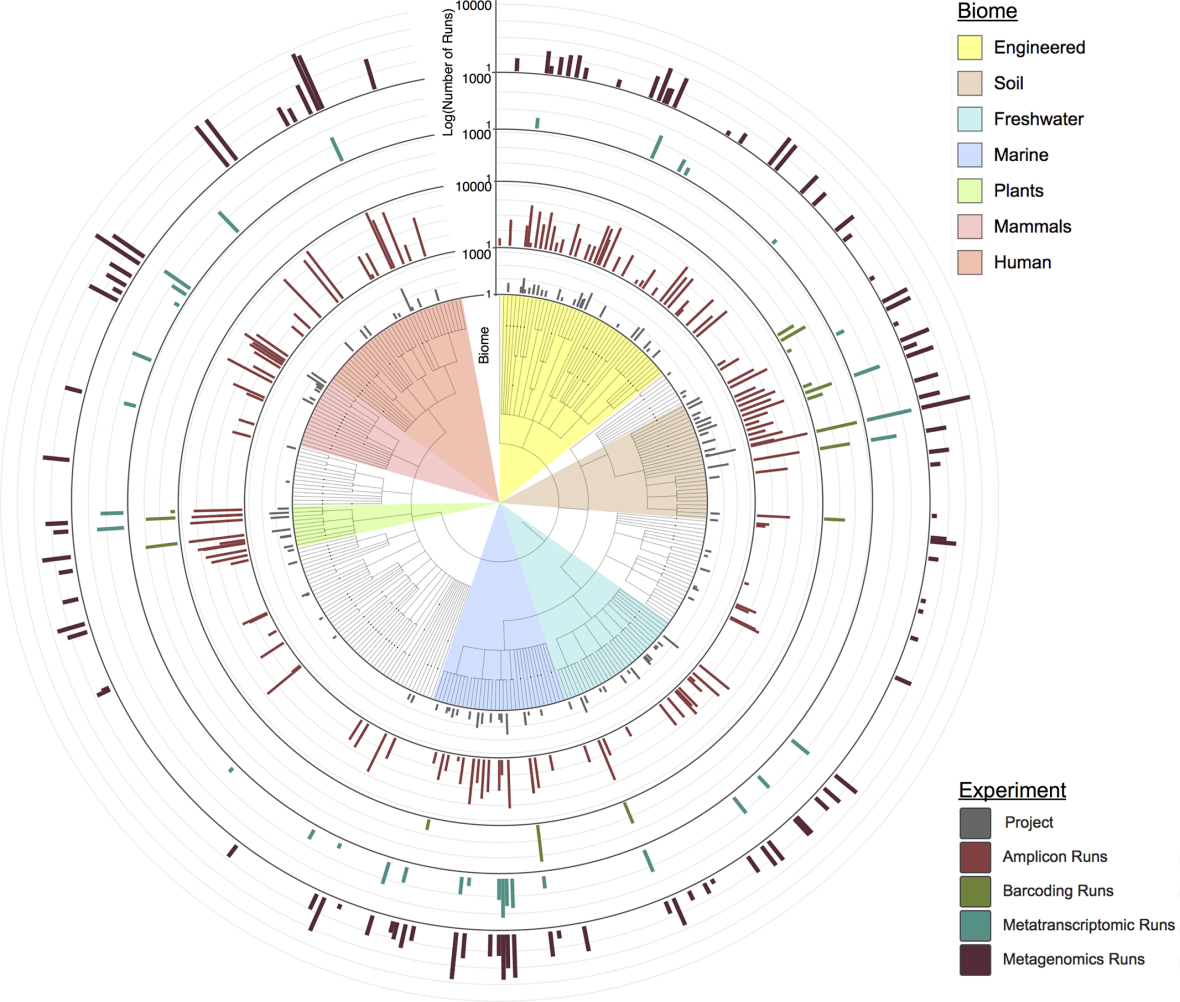
Illustration of the number of projects and runs analysed from each biome. The number of projects and runs from different study types are shown on consecutive log axes. This figure was produced using the iTOL server (44).

EBI Metagenomics currently contains over 1200 publicly available projects, comprising ∼75 000 samples and ∼100 000 runs, representing a 10-fold increase in the number of datasets over the last two years. The majority of this data (∼77 000 runs) are 16S rRNA gene amplicon datasets, followed by WGS metagenomic datasets (∼15 000 runs) with a smaller number of metatranscriptomic studies and assemblies. This is broadly consistent with the breakdown of metagenomic data submission to the ENA. The number of runs for each study type, split according to source environment (also known as ‘biome’), is shown in Figure 1.

## PIPELINE UPDATES

**Figure 2.**
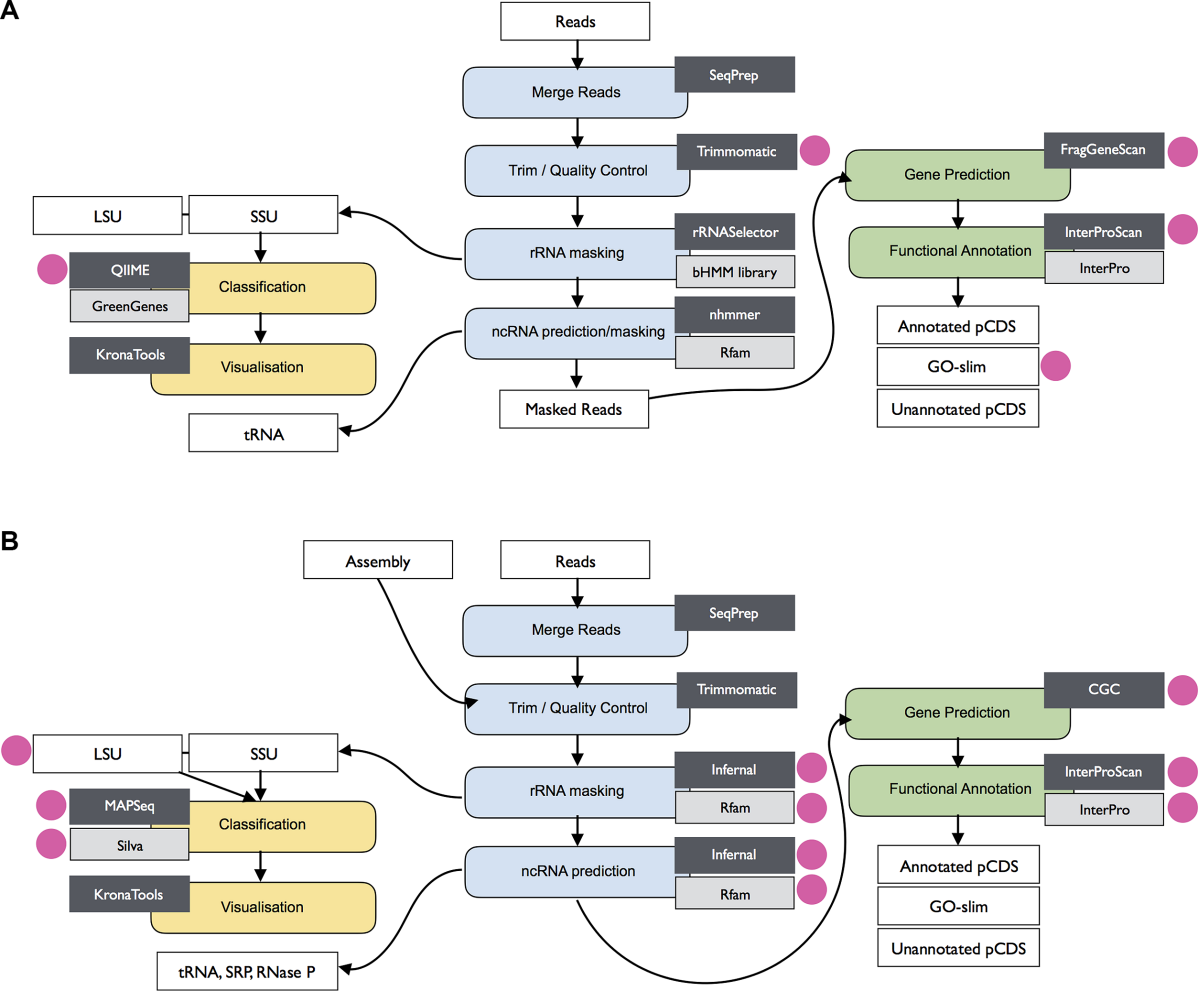
Schematic representations of the EBI metagenomics pipeline versions 3.0 (**A**) and 4.0 (**B**). Tools and reference databases updated in each release are indicated by a magenta circle and described in detail within the text. Processing steps are indicated in the colour rounded boxes (yellow, blue, green), tools in dark grey boxes and databases in light grey boxes. Input and output files as white squares. The combined gene caller component is indicated as CGC.

The EBI Metagenomics team continually evaluates new tools and approaches for potential inclusion in the pipeline with the aim of providing the best possible analyses. An updated version of the analysis pipeline (version 3.0, outlined in Figure 2A) was released in July 2016, with updates to a number of tools and algorithms: InterProScan 5.19 (based on InterPro release 58.0) (20); Trimmomatic 0.35 (21); FragGeneScan 1.20 (13), QIIME, 1.9.1 (22) and a new Gene Ontology slim for visualization (23,24). The QC steps of the pipeline were also extended to cover additional metrics, such as distribution of read lengths, % GC content and relative nucleotide abundances. In addition to enriching the data outputs, this also brought the QC visualizations on the site into closer alignment with those provided by MG-RAST (18), providing consistency and helping users transition from one site to the other.

The analysis pipeline underwent a more substantial update in August 2017 to version 4.0 (Figure 2B), with the entire taxonomic profiling section replaced. The rRNASelector based component (25), which was previously used to identify 16S rRNA genes, was replaced with Infernal (26) (running in HMM-only mode) using a library of ribosomal RNA models from Rfam 12.2 (27) (families comprising Rfam clans CL00111 (SSU) and CL00112 (LSU)). This allows accurate identification of both large and small subunit (LSU and SSU) ribosomal ribonucleic acid genes, including the eukaryotic 18S rRNA gene. In addition to the ribosomal subunit RNAs, the pipeline also extracts other non-coding RNAs (ncRNAs) such as SRP RNA, tRNA, tmRNA and RNase, using the following libraries: CL00001 (tRNA clan), CL00002 (RNase P clan) and CL00003 (SRP RNA clan).

The QIIME taxonomic classification component of the analysis pipeline was replaced with MAPseq version 1.2 (28), which offers fast and accurate classification of reads, and provides corresponding confidence scores for assignment at each taxonomic level. The Greengenes reference database (29) was replaced with SILVA SSU/LSU version 128 (30), which includes eukaryotic as well as prokaryotic sequences, thus enabling eukaryotic taxonomic classification. In order to make it compatible with MAPseq, the SILVA database was remapped to an 8-level taxonomy, using in house scripts. The resulting classification system was compared to QIIME/Greengenes and benchmarked using both mock community and real-world datasets to confirm validity of results.

Prodigal (31) version 2.6.3 was added to run alongside FragGeneScan version 1.20 as part of a combined gene caller component. For assembled sequences, the predictions from Prodigal are supplemented by any non-overlapping regions called by FragGeneScan. For short reads, FragGeneScan alone is used. Finally, InterProScan was updated to version 5.25 (based on InterPro release 64.0).

## EXTENDED TAXONOMIC ANALYSIS TO ALL CELLULAR LIFE

**Figure 3.**
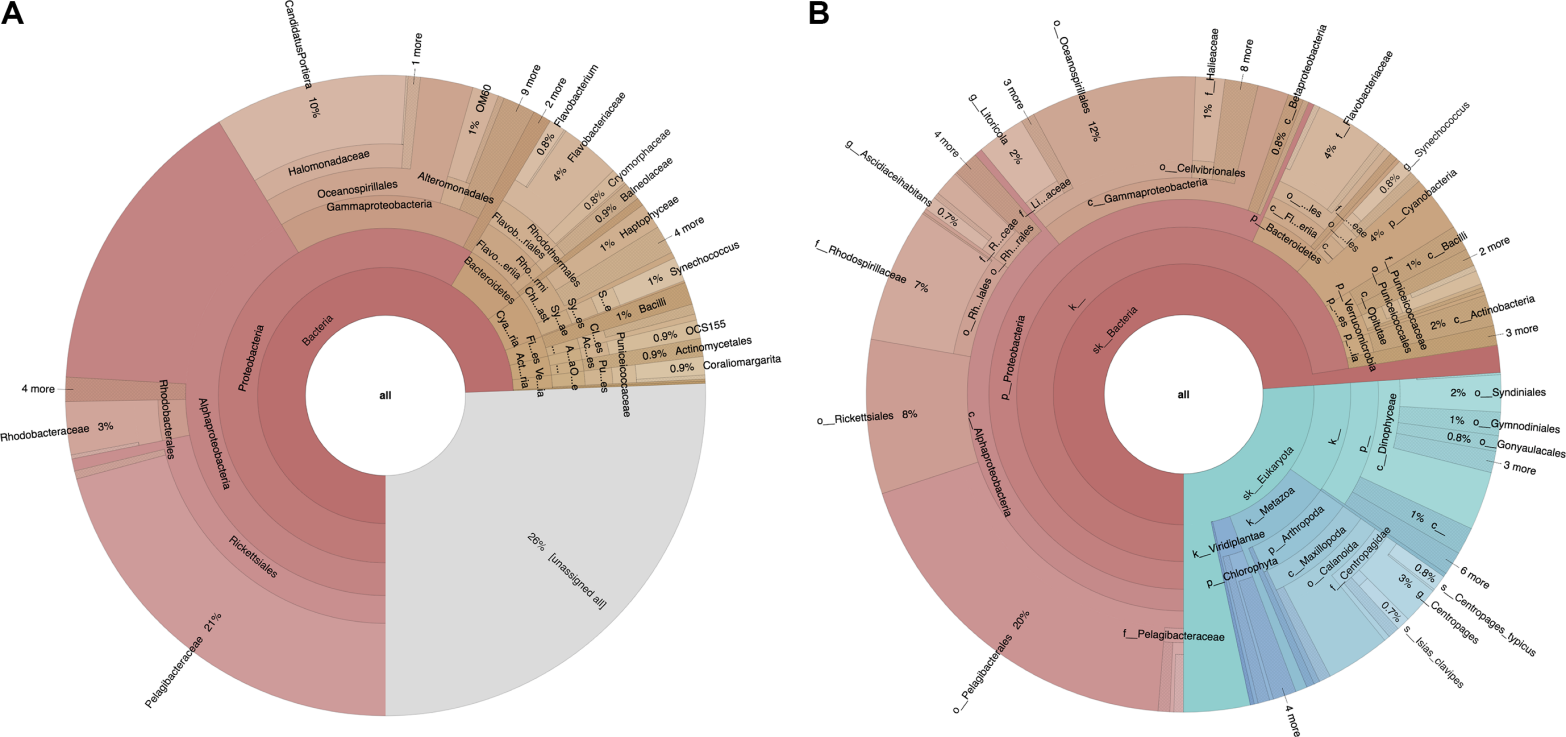
Krona plots showing taxonomic classification of run ERR771104 from Ocean Sampling Day 2014 (ENA project accession PRJEB8682). (**A**) Produced using version 2.0 of the pipeline and (**B**) using version 4.0. Prokaryotic taxonomic lineages are shown in red, eukaryotic in blue and unclassified in grey. The total number of 16S rRNA/SSU input sequences was similar in each case (976 with version 2.0 versus 1008 with version 4.0).

The adoption of Infernal using a comprehensive library of ribosomal models means that the pipeline now identifies SSUs (16S and 18S rRNAs). Comparing these against the SILVA database that includes both prokaryotic and eukaryotic references allows rich classification and a reduction in the number of sequences labelled as unclassified (see Figure 3).

## UPDATE OF THE METAGENOMICS GO SLIM

As part of the pipeline 3.0 update, the metagenomics GO slim, which is used to summarize functional annotations for visualization on the website, was rebuilt. This was based upon the analysis of all GO terms that had been assigned to approximately 20 billion protein coding sequences using pipeline version 2.0. The updated slim provides summarization for 97% of terms, compared to 83% previously, and therefore gives a better representation of the underlying annotations. The updated metagenomics GO slim is available at: http://www.geneontology.org/ontology/subsets/goslim_metagenomics.obo.

## DATA DISCOVERY AND RETRIEVAL INTERFACES

With the rapid expansion in the number of datasets, it has become increasingly important to improve access to the data contained within EBI Metagenomics for exploration and discovery. To this end, we have made use of the EMBL-EBI search (32) infrastructure to implement more powerful browsing and searching. A search input box is present on all pages, allowing entry of free text (eg, ‘human’) or colon-separated fields and values (eg, ‘experiment_type:amplicon’). Searches are subdivided into three levels: projects, samples and runs, as each level has different metadata available. The results are displayed in separate tabs and can be filtered by facets and numerical search controls, as appropriate for the data type. For example, run-level has the richest set of indexed facets that can be used for filtering, with Organism, GO-terms and InterPro annotations. The latter two can also be used as search terms, and the results can then be filtered by fields such as temperature or depth. Using this search interface, it is possible to rapidly and easily narrow down datasets (for example, to discover all runs that contain antibiotic biosynthesis monooxygenase sequences in soil, where Actinobacteria are found, determined using metatranscriptomics).

To provide a richer search and retrieval interface, we have begun development of a RESTful API, providing programmatic access to the data. The base address to the API is https://www.ebi.ac.uk/metagenomics/api/v0.2/. There are several top-level resources, such as studies, samples, runs, experiment-types, biomes and annotations. Links to a resource (eg, https://www.ebi.ac.uk/metagenomics/api/v0.2/biomes) return a JSON object formatted data structure that contains the resource *type* (in this example ‘biomes’), associated object identifier (*id*) and *attributes*. Where appropriate, *relationships* and *links* are provided to other resources, allowing complex queries to be constructed. For example, https://www.ebi.ac.uk/metagenomics/api/v0.2/studies retrieves a list of all studies, while https://www.ebi.ac.uk/metagenomics/api/v0.2/studies/ERP009004 retrieves a single study, with the accession ERP009004. The samples contained within this study can be retrieved using the following URL: https://www.ebi.ac.uk/metagenomics/api/v0.2/studies/ERP009004/samples.

**Figure 4.**
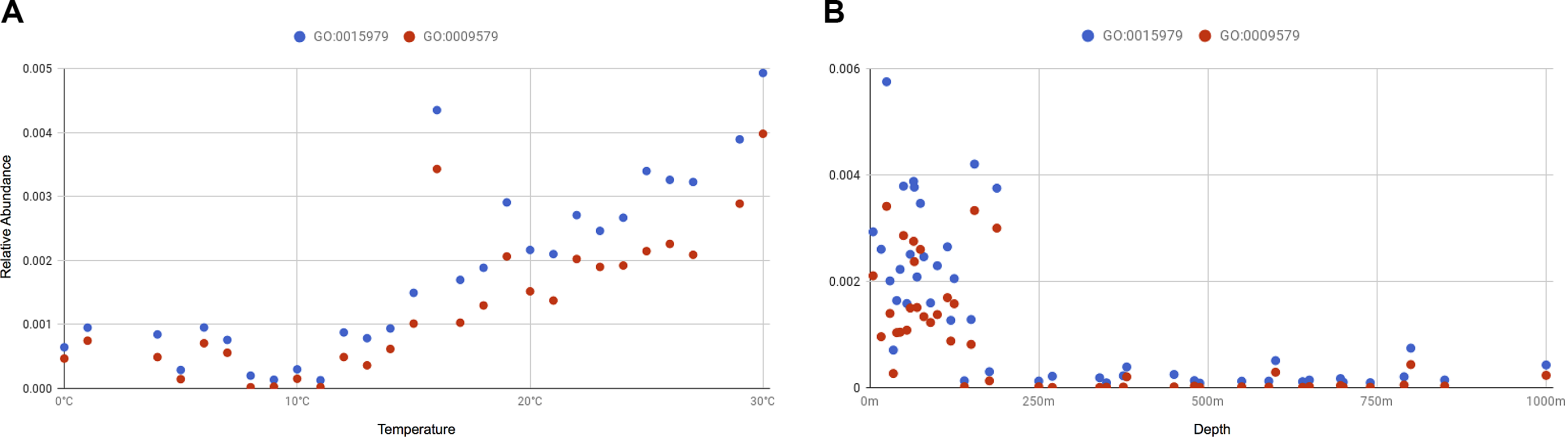
Correlation between temperature (**A**) and depth (**B**) and photosynthesis-related GO term counts, normalized by number of InterPro annotations, for Tara Oceans project PRJEB1787. Metadata and annotations were retrieved from the API and combined on the fly to generate the visualizations.

Lists of resources can be filtered and sorted by selected *attributes*, allowing the construction of more complex queries. For instance, in order to retrieve oceanographic samples from metagenomic studies taken at temperature less than or equal to 10°C, the following query could be constructed https://www.ebi.ac.uk/metagenomics/api/v0.2/biomes/root:Environmental:Aquatic:Marine/samples?experiment_type=metagenomic&metadata_key=temperature&metadata_value_lte=10&ordering=accession. The provision of such complex queries allows metadata to be combined with annotation for powerful data analysis and visualisation (see Figure 4).

As some queries can result in a large response, the API supports pagination, using a page number and size of results per page as query parameters. The API response also distinguishes between *attributes* and *relationships*, allowing customization of the response by passing fields or including relations as parameters in the initial query. We have utilized an interactive documentation framework (Swagger UI) to visualize and simplify interaction with the API’s resources via an HTML interface. Detailed explanations of the purpose of all resources, along with many examples, are provided to guide end-users. Documentation on how to use the endpoints is available at https://www.ebi.ac.uk/metagenomics/api/docs/.

## DIVERSITY ESTIMATION AND SAMPLE COMPARISONS

As part of pipeline v4.0, we have added additional information regarding metagenomic community diversity estimation, and information to allow comparisons between runs and samples. For each run and sample we produce plots to graphically illustrate the taxa abundance distribution, and use OTU counts to compute diversity indices, including several estimates of the total diversity of the community sampled. These estimates are computed using R packages for community ecology such as *sads* (https://CRAN.R-project.org/package=sads) and *vegan* (https://CRAN.R-project.org/package=vegan). Estimates are also computed at the level of sample, based on simple pooling of OTU counts from all runs in a sample. Additionally, estimates are provided for the number of individuals that would need to be sequenced in order to see a given fraction of the total population diversity (based on the assumption of an underlying Poisson-log-normal taxa abundance distribution). These provide guidance for the sequencing effort likely to be required for a more complete characterisation of the microbial community of interest.

Additional diagnostic plots have also been added which allow for the comparison of samples and runs within a study. In addition to a PCA plot for the identification of outlying runs, these provide a robust estimate of the fold-change difference in taxonomic composition between a reference sample (or run) and all other samples (or runs) in the study. Estimates of differences are computed using the *DESeq2* (33) software via the Bioconductor package *phyloseq* (34). These estimates are most robust for studies with replication in the form of multiple runs per sample.

## ASSEMBLY AND PEPTIDE DATABASE CREATION

In 2017, we undertook a feasibility study aimed at investigating assembly of metagenomic datasets given current infrastructural resources. During this study, we evaluated a number of different assembly algorithms with respect to processing speed and memory usage, as well as quality of assemblies. Based on the results, we believe it is feasible to offer assembly of user-submitted metagenomic datasets, subject to request. We have chosen a panel of three assemblers for use with the pipeline: metaSPAdes, MEGAHIT (35) and Minia (36). This selection is based on the quality of assembly across a range of biomes and the resources required to produce an assembly.

MetaSPAdes and MEGAHIT have been found to be amongst the best performing assemblers for metagenomic data, according to independent review (14). Minia, meanwhile, has very low memory requirements and can be used to assemble very large datasets from diverse communities. These assertions have also been confirmed by other benchmarking initiatives, such as CAMI (http://dx.doi.org/10.1101/099127), which also demonstrated that Minia excelled at assembling abundant circular elements.

To enable the selection of the optimal assembly pipeline (for a given data set and available compute resources) we have developed a neural network, built using TensorFlow (https://arxiv.org/abs/1603.04467). This considers inputs such as source biome, sequencing platform, file size, read count and base count to estimate and assign the appropriate assembler and memory parameters. metaSPAdes is used as the default, with Minia as the alternative if predicted metaSPAdes memory requirements are too high. MEGAHIT, which offers a middle ground in term of memory usage, can be used in place of metaSPAdes, if requested by users.

As part of the assembly tool evaluation process, we assembled a number of publicly available metagenomic datasets from ENA, drawn from a diverse range of environments. To date, we have assembled 2,298 different shotgun metagenomics datasets from 78 different projects. All bar nine of these have been assembled using metaSPAdes, with eight assembled using Minia and one using MEGAHIT. Of the assemblies, 1,935 are from a range of human microbiome projects, including the HMP project (37) that was previously absent from EBI Metagenomics. These are accompanied by smaller numbers of assemblies from a range of biomes: 187 marine, 151 from soil, 10 wastewater sludge, 6 freshwater, 7 animal gut microbiomes and 2 others. As EBI Metagenomics has remits to both analyse and archive, these assemblies have also been submitted to the ENA (accessions summarised in Supplementary Table S1), before being retrieved for analysis with the EBI Metagenomics pipeline. Combined with the pre-existing assemblies, EBI Metagenomics now contains over 2400 assembled datasets, corresponding to 13% of the shotgun metagenomics data. Having established that it is possible to offer assembly as an analysis service, we will continue to assemble public datasets internally and respond to user request to assemble other datasets (either their own private data or public data) until our user interfaces are enriched to allow the selection of the analysis type directly.

**Figure 5.**
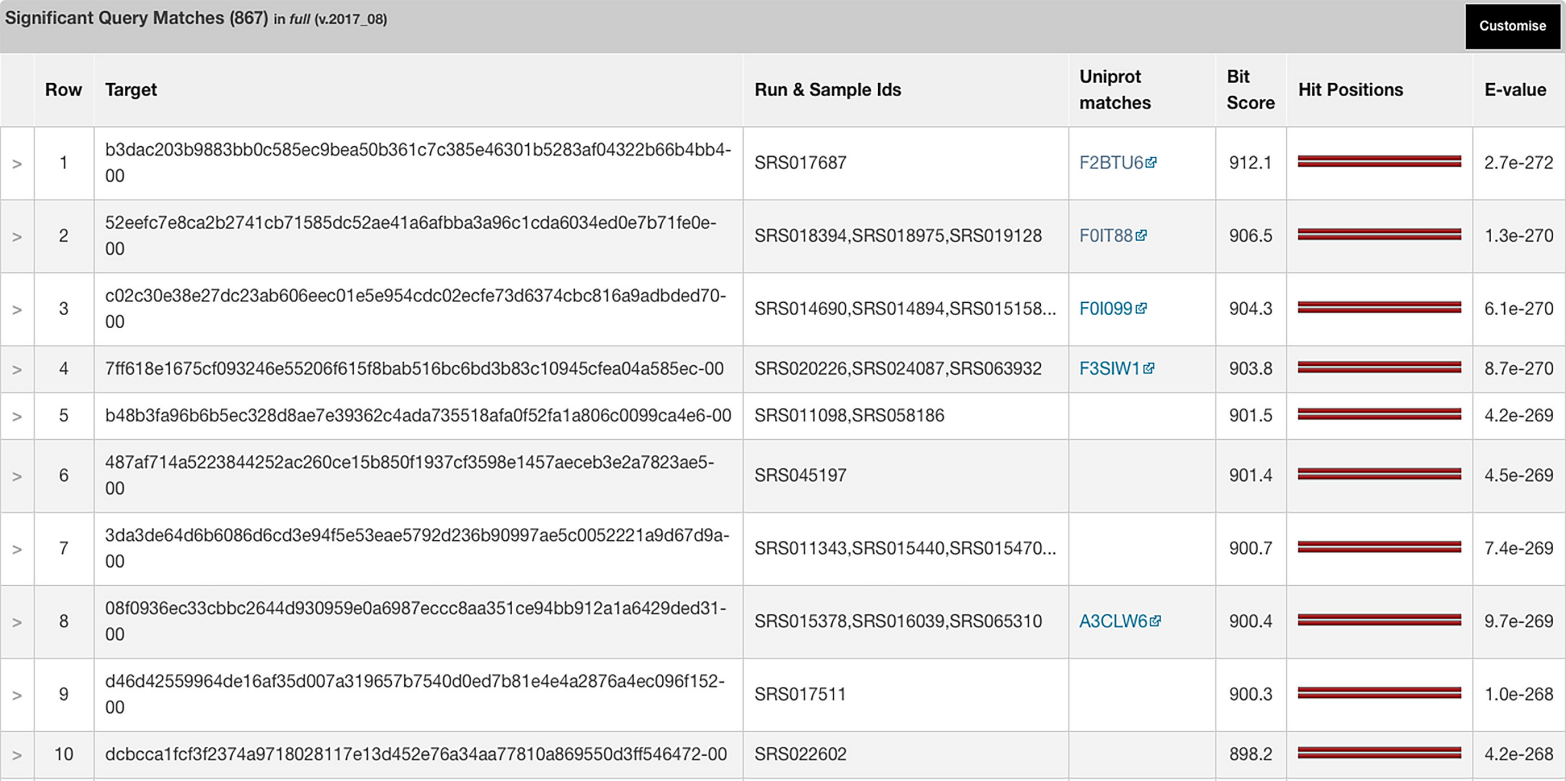
HMMER search results using the assembled peptide database. Searching the full length subdivision of the assembled peptide database with an arginine deiminase from *Streptococcus sanguinis SK1057* (UniProt identifier: F2BTU6) identified over 800 sequences with a significant match (*E*-value < 1e–10) to the query sequence, with <9% (78 sequences) having an identical counterpart in UniProtKB.

As a compendium to the assemblies and their associated analysis results, we have developed a workflow to produce a non-redundant set of peptides. From an initial 400 assembly datasets, a non-redundant peptide database of almost 50 million sequences has been produced. Over 15 million of these are predicted to be full length, yet only ∼1 million have exact counterparts in the UniProtKB (38) database. To allow the querying of this sequence database by users with a target sequence, we have deployed a HMMER web search engine and server (39). This interface can be accessed via a tab on the front page of the website. Sequences can be queried against either the entire database, or just the full-length sequences, or the fragment subset. Matching sequences are displayed, along with alignments between the query and matches, and cross-links to any matching counterparts in UniProtKB (see Figure 5). Sequences are also mapped to the runs and/or samples from which they originated, allowing contextual metadata to be associated. We expect this database to grow rapidly, reaching 100s of millions, or even billions, of sequences as we assimilate more and more proteins from our assemblies.

## DATA SUBMISSION SERVICES AND GROWTH

Data submission to EBI Metagenomics is routed via ENA, which offers interactive and programmatic interfaces, supported by an active submissions helpdesk and training materials. There have been incremental updates to this service through 2017, including improvements to sample description and assembly submission user workflows. From its inception, EBI Metagenomics has had a commitment to supporting data standards, to allow full discoverability, interoperability and reusability of data. Focusing especially on contextual data standards around sample descriptions, which are essential in interpreting metagenomics studies, ENA implements the MIxS standards in its submission system (40). ENA has continued to track developments in MIxS and its underlying structured vocabularies. A third of metagenomics samples submitted through EMBL-EBI see users appropriately selecting MIxS sample checklists during the submission process. An ongoing discussion with INSDC (41) partners assures uptake of these standards for those datasets not routed through submissions services at EMBL-EBI.

**Figure 6.**
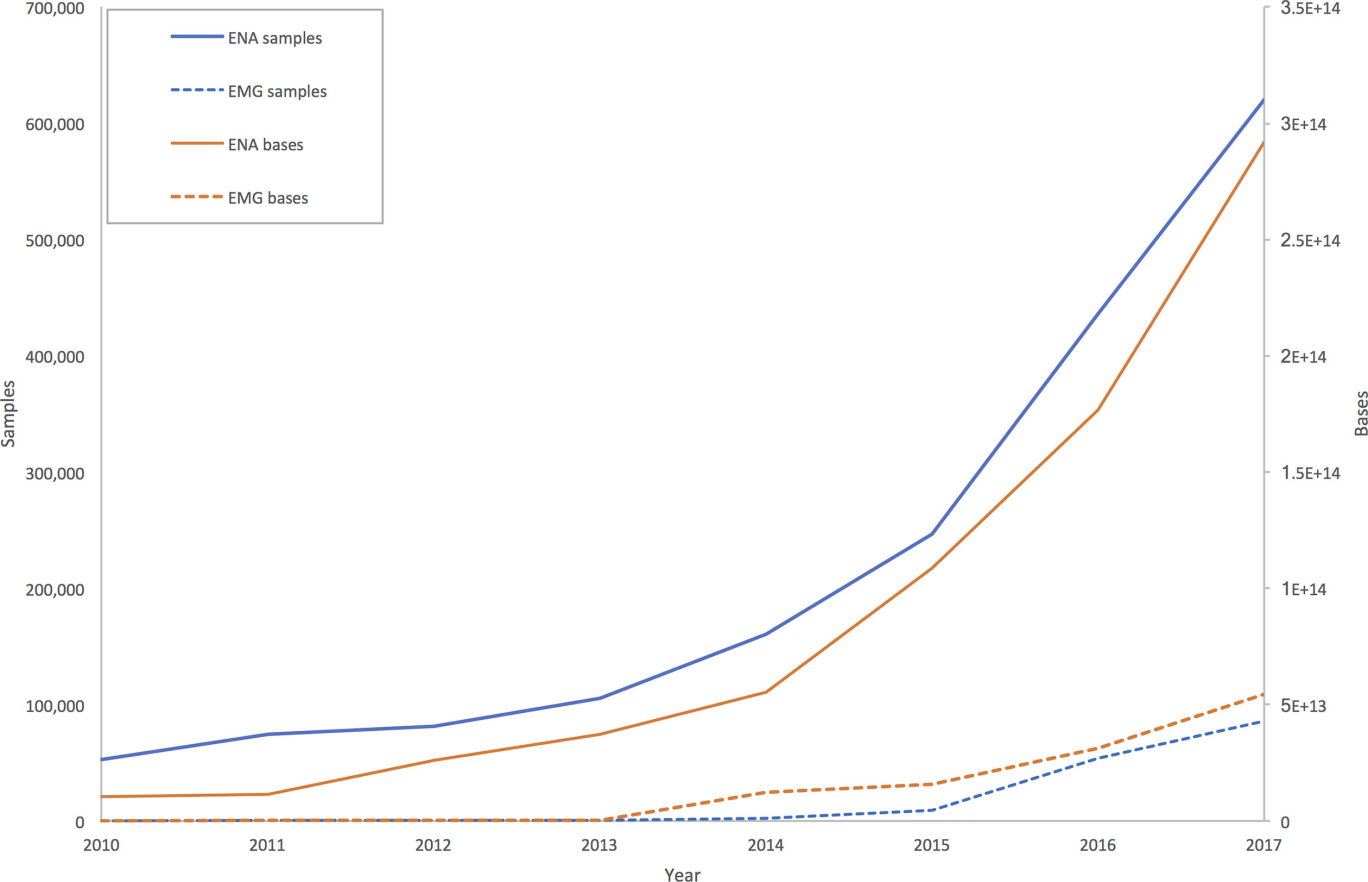
Growth of metagenomics data housed in ENA and processed by EBI Metagenomics (EMG). This graph shows the cumulative growth of environmental data in the two resources (ENA: solid lines, EMG: dashed lines) according to two different metrics: numbers of samples (blue) and number of bases (orange).

The rate of growth of metagenomic sequence submission to ENA is increasing, as illustrated in Figure 6. At the same time, the rate of data analysis by EBI Metagenomics has also increased following a major pipeline improvement in 2014 aimed at optimising analysis throughput. While a sizable volume of data has now been analysed, this represents only a small proportion of overall available data, and further pipeline improvements will be required to keep pace. However, not all of the data identified within the ENA will be tractable for analysis. For example, some samples are metabarcoding studies targeting specific marker genes outside of the scope of the pipeline. Others are isolate genomes from retrieved from an environmental sample, which is also outside of the current scope of the resource.

## IMPROVED DOCUMENTATION

Over the past two years our range of services has changed and expanded substantially. While we endeavour to inform our user community via blogs or social media, information disseminated in this way can be transient. In the past, our help pages have been relatively brief and lacked search functionality. To overcome these deficiencies, we now provide revised and expanded documentation for both the pipeline and website, hosted externally at Read the Docs (http://emg-docs.readthedocs.io/). The advantages offered by this service include the ability to search within documentation, to export the documents in numerous formats and to support concurrent versions for different pipeline/website releases. Furthermore, as this documentation is available in GitHub, it allows team members, external collaborators and users to contribute to the documentation

## DISCUSSION

As the field of metagenomics develops, data volumes grow, and sequence processing and analysis algorithms mature, it is important that analysis pipelines evolve to keep pace. EBI Metagenomics began life several years ago as a small resource, largely devoted to processing Roche 454 metagenomic data (42). It has grown to become one of the largest metagenomic repositories in the world, supporting the analysis of a range of study types, generated with a variety of different sequencing technologies, from a range of different biomes. The next stage in its development has been to extend and improve its processing and analyses to keep up with progress in the field. At the same time, it has looked to extend access to captured contextual metadata and analysis results, to become more useful to the research community.

To this end, the last two years have seen EBI Metagenomics pipeline changes aimed at updating tools and broadening the scope of analyses. These have included new components to enable eukaryotic taxonomic analysis and better representation of functional annotation using updated reference databases. At the same time, extensive data indexing to support web-based searches and a new RESTful API serving contextual metadata and results have been developed to offer powerful entry points for browsing, searching and discovery of data from both a manual and programmatic perspective.

A more fundamental change is the shift towards provision of assembly of both user-submitted and publicly available metagenomic datasets. This represents a new and exciting development for the resource, which will provide the opportunity to compare the differences between raw reads and assembly analysis outputs (a comparison that will require tracking of raw reads to contigs to maintain abundance counts).

Assembly also opens the door to more in-depth functional annotation. For example, provision of full length protein sequences potentially allows their annotation using the complete set of InterPro (43) member databases, some of which are excluded from the current EBI Metagenomics pipeline as they do not perform well at annotating sequence fragments. The ability to annotate at a very deep functional level (e.g. classifying sequences into specific protein subfamilies and identifying precise enzymatic functions) in turn allows more sophisticated analyses, such as reconstruction of precise metabolic pathways within a microbial community at very high resolution. Furthermore, assembly also unlocks the possibility of taxonomic binning, genome reconstruction and annotation, which brings the potential for identification of new organisms.

Such developments will provide new analysis types and entry points to the resource. The first of these is already accessible in the form of the nascent non-redundant peptide database, described above. The generation of such a database—and the ability to run sequence-based searches against it—addresses a feature frequently requested from our user community. Other entry points or views of the data, such as intuitive visualisations of assembled contigs and their associated annotations, are currently lacking and will need to be developed.

As EBI Metagenomics continues to evolve, careful thought will need to be given as to how new features scale, to balance usability with sustainability. For example, generating one vast peptide database based on all assembled sequence data will not address community needs, as the data needs to be structured in meaningful ways (for example, mapping the sequences back to particular biomes and/or environmental conditions). At the same time, we will need to monitor our assembly strategy, since without algorithm development, there will be many projects where it is simply computationally too expensive to assemble all sequence data without impacting analysis of user-submitted data and the day-to-day activities of the resource. Dealing with these issues, and with those outlined above, will be a key priority for EBI Metagenomics in the forthcoming years.

## AVAILABILITY

All of the assemblies produced by this work have been submitted to the ENA. Accession numbers are given in Supplementary Table S1.

## Supplementary Material

Supplementary Data
