# Supplementary material for: EBI Metagenomics in 2017: enriching the analysis of microbial communities, from sequence reads to assemblies
Source: Nucleic Acids Res. 2017 Oct 23;46(Database issue):D726–35. doi: 10.1093/nar/gkx967 (PMC5753268; doi:10.1093/nar/gkx967)
Supplement: Supplementary Data [file gkx967_supp.pdf]

Sample biome	Sub-biome	Project identifiers	Number of assemblies
Aquatic	Freshwater	*PRJEB22523, PRJEB22543, PRJEB22524*	*4*
	Glacier	*PRJEB22516*	*3*
	Lentic	*PRJEB22499*	*1*
	Sediment	*PRJEB22502, PRJEB22517, PRJEB22496*	*7*
	Marine	*PRJEB22527, PRJEB22092*	*142*
	Coastal	*PRJEB22525, PRJEB22509*	*5*
	Hydrothermal vents	*PRJEB22510, PRJEB22514, PRJEB22397*	*14*
	Salt marsh	*PRJEB22494*	*1*
	Oceanic	*PRJEB22493, PRJEB22522*	*9*
	Photic zone	*PRJEB22394*	*11*
	Alkaline	*PRJEB22526*	*1*
Engineered	Activated sludge	*PRJEB22508, PRJEB22512, PRJEB22550, PRJEB22521*	*10*
Host-associated	Human	*PRJEB22283, PRJEB22387, PRJEB22388, PRJEB22556*	*821*
	Human, faecal	*PRJEB22492, PRJEB22505, PRJEB22513, PRJEB22495, PRJEB22501, PRJEB22498, PRJEB22392, PRJEB22359, PRJEB22360, PRJEB22519, PRJEB22529, PRJEB22395, PRJEB22538, PRJEB22365, PRJEB22542, PRJEB22391, PRJEB22547, PRJEB22549, PRJEB22389, PRJEB22393, PRJEB22552, PRJEB22368, PRJEB22554, PRJEB22362*	*1108*
	Human, large intestine	*PRJEB22528*	*1*
	Human, oral	*PRJEB22520*	*1*
	Human, skin	*PRJEB22555*	*5*
	Rumen	*PRJEB22504, PRJEB22623*	*6*
Soil	Soil	*PRJEB22518, PRJEB22546, PRJEB22551, PRJEB22533, PRJEB22511, PRJEB22503, PRJEB22544, PRJEB22540, PRJEB22396, PRJEB22366, PRJEB22627, PRJEB22625, PRJEB22624*	*129*
	Agricultural	*PRJEB22531*	*1*
	Forest soil	*PRJEB22506*	*5*
	Grasslands	*PRJEB22497, PRJEB22500, PRJEB22628*	*5*
	Permafrost	*PRJEB22530*	*4*
	Sand	*PRJEB22545*	*3*
	Wetlands	*PRJEB22541*	*1*

Supplementary Table 1. Distribution of EBI Metagenomics assemblies according to source biome. The table lists the source biome, sub-biome (where available) and ENA identifiers of projects that have been assembled and analysed by EBI Metagenomics. The total number of assemblies for each sub-biome is also shown.
